# The application of wing-shaped window decompression device in the treatment of large odontogenic keratocyst: An observational study

**DOI:** 10.1097/MD.0000000000039601

**Published:** 2024-09-06

**Authors:** Haifeng Liu, Nuo Zhou, Xuanping Huang

**Affiliations:** a Department of Oral and Maxillofacial Surgery, College of Stomatology, Hospital of Stomatology, Guangxi Medical University, Nanning, China; b Guangxi Key Laboratory of Oral and Maxillofacial Rehabilitation and Reconstruction, Guangxi Medical University, Nanning*,* China; c Guangxi Clinical Research Center for Craniofacial Deformity, Guangxi Medical University, Nanning, China.

**Keywords:** border molding, decompression, odontogenic keratocysts, plug

## Abstract

To enhance the decompression and drainage effects after marsupialization of large odontogenic keratocysts (OKCs) in the jawbone, a novel cyst plug was designed, and its clinical feasibility was investigated. A total of 42 patients with large OKCs requiring decompression were divided into 2 groups: the control group (n = 21), which underwent traditional drainage tube insertion, and the experimental group (n = 21), which received a personalized wing-shaped plug. The clinical efficacy of the wing-shaped plug was assessed, and postoperative recovery times were compared between the 2 groups. The average duration of use for the wing-shaped plug was approximately 14 months. Compared to the control group, the treatment duration in the experimental group was reduced by about 3 months. The wing-shaped plug demonstrated superior fit, comfort, reduced food residue, minimal irritation to surrounding tissues, a more aesthetically pleasing appearance, and less reported pain. The adaptive wing-shaped plug offers improved prognosis for patients undergoing decompression of OKCs and shows significant potential for clinical application.

## 1. Introduction

Odontogenic keratocyst (OKC) is a common cystic benign lesion of the jawbones, previously believed to exhibit locally aggressive growth and a tendency for postoperative recurrence, and was thus considered a tumor.^[1,2]^ However, with a deeper understanding of the biological behavior of this disease, the World Health Organization (WHO) reclassified OKC as a cyst in the latest (5th) edition of the WHO Classification of Head and Neck Tumors released in May 2022.^[3]^ This reclassification signifies a shift in the surgical treatment concept for OKC from radical to a combination of radical and functional approaches. Specifically, for initial, localized OKCs without high-risk factors for recurrence, enucleation is the preferred treatment.^[4,5]^ However, for large cystic lesions of the jaw involving multiple adjacent teeth, the inferior alveolar nerve canal, maxillary sinus, or nasal cavity, or for children and adolescents with cysts containing functional teeth, marsupialization is recommended first, followed by enucleation after the cyst has reduced in size to achieve definitive treatment.^[4,5]^ Combining radical and functional treatment approaches is crucial for adequately protecting the anatomical structures of the patient’s oral cavity, actively maintaining physiological function, and improving quality of life.^[4–6]^

Marsupialization involves keeping the cavity of large OKCs open using decompression devices, reducing intracystic pressure and stimulating adjacent bone regeneration, thereby gradually shrinking the cyst.^[7]^ Traditional decompression devices mostly use drainage tubes, such as those proposed by Kolokythas, who placed a drainage tube in the cyst cavity and fixed it to adjacent teeth with wires.^[8]^ Swantek et al recommended suturing the drainage tube placed in the cyst cavity to the surrounding soft tissue.^[9]^ These methods are often difficult to clean and may cause pain, discomfort, tissue overgrowth, or even displacement or blockage of the drainage tube, affecting efficacy.^[7]^ Recently, some scholars have suggested tooth-supported decompression devices similar to removable dentures, which allow patients to remove and clean the cyst cavity freely.^[10]^ However, these devices have drawbacks such as large size and discomfort, and are especially unsuitable for patients without missing teeth.^[10]^

Therefore, this study proposes a self-adaptive personalized “wing-shaped decompression device” based on impression and border molding techniques. It prospectively analyzes the clinical outcomes and patient comfort during marsupialization treatment of large OKCs using this device, and conducts a randomized controlled trial comparing it to the traditional drainage tube decompression method to evaluate the clinical feasibility of this new device.

## 2. Materials and methods

### 2.1. Study subjects

This study included patients with large OKC of the jawbones treated in the Department of Oral and Maxillofacial Surgery at the Affiliated Stomatological Hospital of Guangxi Medical University from January 2020 to December 2021. All procedures involving human participants in this study complied with the Helsinki Declaration (revised in 2013). The study was approved by the Ethics Committee of the Affiliated Stomatological Hospital of Guangxi Medical University (Approval No.: 2021021). Informed consent was obtained from all the participants or their guardians.

### 2.2. Inclusion and exclusion criteria

#### 2.2.1. Inclusion criteria were as follows

1. Pathological diagnosis of odontogenic keratocyst.
2. No history of jaw cysts.
3. Ability to comply with follow-up.
4. Suitable candidates for marsupialization and willingness to participate with signed informed consent.
5. Cyst volume >4 cubic centimeters, involving at least 1 of the following anatomical structures: teeth, inferior alveolar nerve canal, maxillary sinus, nasal cavity, or functional tooth buds.

#### 2.2.2. Exclusion criteria were as follows

1. Patients with contraindications for surgery.
2. Unsuitable candidates for marsupialization.
3. Refusal to participate or sign informed consent.
4. Noncompliant with follow-up.

### 2.3. Surgical procedure

**Figure 1.**
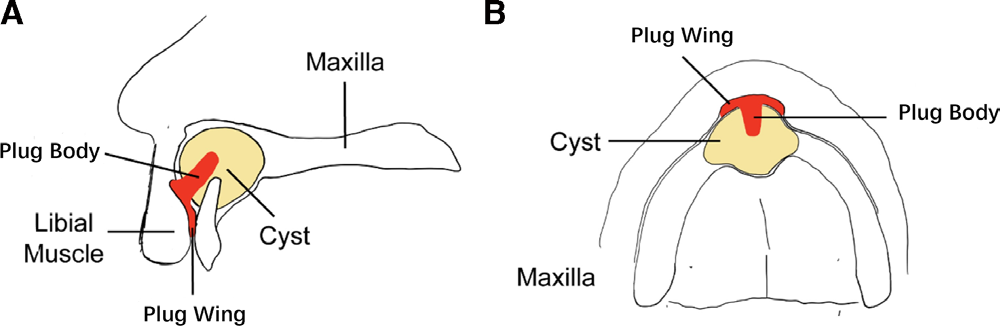
Schematic diagram of the wing-shaped plug.

Patients with large odontogenic keratocysts who met the inclusion criteria were divided into the experimental group (group 1) or the control group (group 2) based on voluntary participation. Group 1 underwent marsupialization with the “wing-shaped decompression device” proposed in this study (Fig. 1), while group 2 received the traditional drainage tube method.

#### 2.3.1. Group 1 procedure

**Figure 2.**
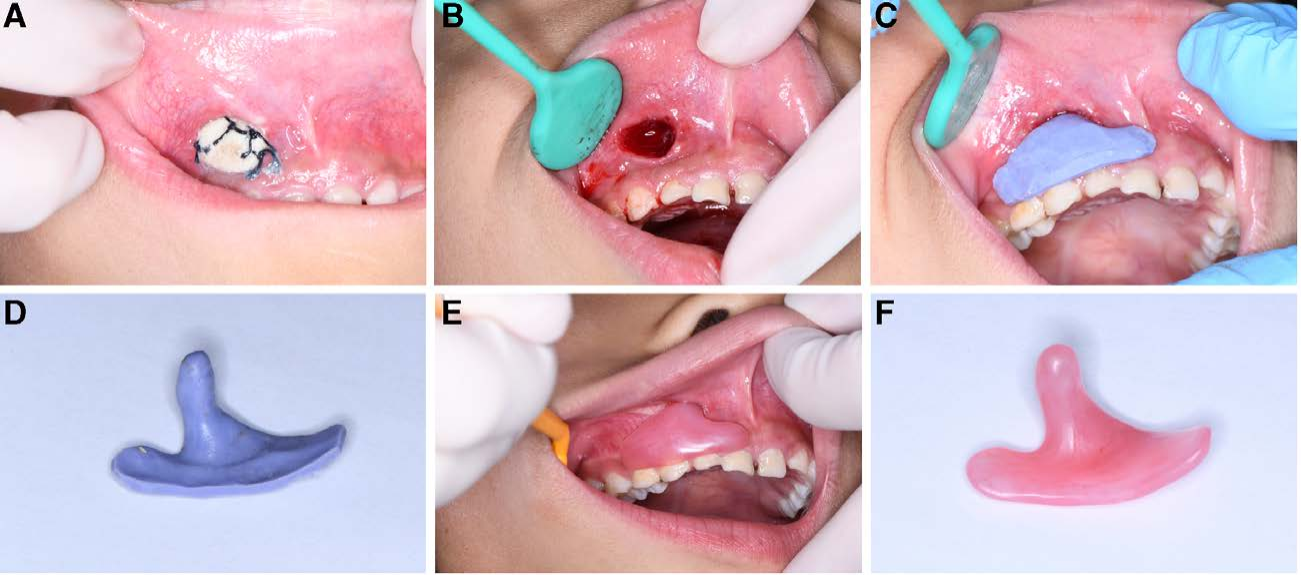
(A) Columnar iodoform yarn was packed into the opening window. (B) Remove the columnar iodoform yarn. (C) Use impression materials to make the plug model. (D) In view of silicone rubber. (E) Wear the final cyst plug into the patient’s oral. (F) The finished cyst plug.

A full-thickness tissue specimen (1 × 1 cm) was excised from the vestibular sulcus, including mucosa, periosteum, cortical bone, and cyst wall tissue, creating a surgical window. This step also served as a biopsy procedure. A cylindrical gauze impregnated with iodoform (1 cm in diameter, 2 cm in length) was then tightly packed into the window and secured to adjacent soft tissue with sutures to prevent displacement into the cyst cavity (Fig. 2A). After a definitive pathological diagnosis, the gauze was removed (Fig. 2B). Silicone rubber impression material was manually shaped into the preliminary form of the “wing-shaped decompression device” before it hardened. The device was designed with a body part that extended 1.5 cm into the cyst cavity and a wing part that fitted into the vestibular sulcus. The wing was molded using muscle function techniques to ensure it conformed to the vestibular sulcus and surrounding tissues, achieving retention under lip and cheek pressure. Once the silicone rubber hardened, it was removed, and excess material was trimmed so that the wing extended to the roots of 4 teeth, with a thickness of 2 to 3 mm (Fig. 2C and D). The silicone rubber device was then sent to a dental lab to be fabricated using polymethyl methacrylate (Biotones, Denken-highdental Co., Ltd, Japan) (Fig. 2E). Patients wore the “wing-shaped decompression device,” removed it after meals, and irrigated the cyst cavity with saline 3 times daily.

#### 2.3.2. Group 2 procedure

**Figure 3.**
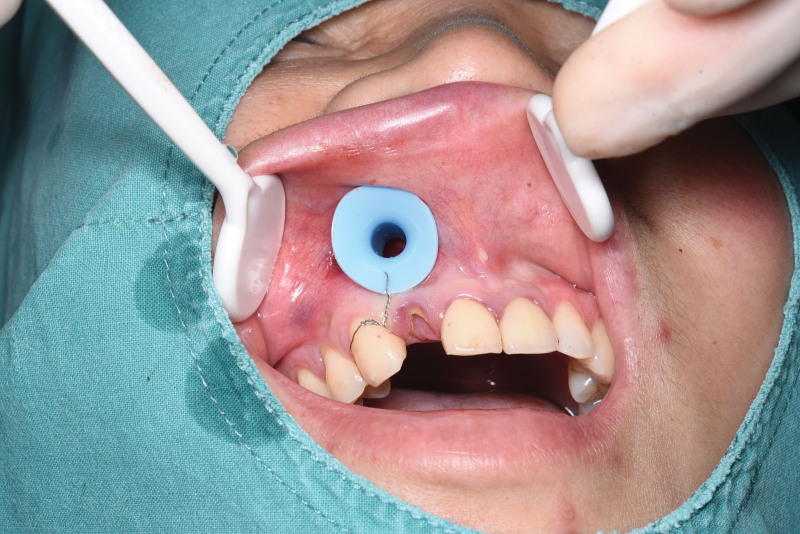
Control group with drainage tube secured by steel wire.

A preformed nasopharyngeal airway tube was cut to approximately 2 cm, inserted into the cyst cavity through the surgical window, and secured to the tooth cervix with stainless steel wire (Fig. 3). Patients irrigated the cyst cavity with saline through the tube 3 times daily, using 60 mL each time after meals.

### 2.4. Clinical efficacy evaluation

Patients in both groups were evaluated for clinical efficacy at 6 months and 12 months. The evaluation was based on subjective and radiographic assessments.

#### 2.4.1. Subjective evaluation criteria

Satisfactory: tissue-friendly, good retention, nondeformable, pain-free, easy to clean, no impact on daily life.

Unsatisfactory: Easy loosening, deformable, difficult to clean with significant plaque accumulation, causing pain, inducing tissue overgrowth, and impacting daily life.

#### 2.4.2. Radiographic evaluation

All patients underwent cone beam CT scans at baseline 0 months, 6 months, and 12 months. Cyst volumes were measured using Mimics Medical 21.0. At the 12-month mark, follow-up appointments and enucleation were scheduled based on the size of the cyst cavity. Enucleation was performed when the cyst volume was reduced to <1 cubic centimeter. The duration from the initial marsupialization to enucleation was recorded for all patients.

### 2.5. Statistical analysis

Statistical analysis was performed using SPSS 25.0. Data were expressed as mean ± standard deviation. Differences between groups were compared using *t*-tests, Chi-square tests, and Fisher exact tests. A *P*-value < 0.05 was considered statistically significant.

## 3. Results

### 3.1. Study population

A total of 42 patients were included in this study, all diagnosed with OKC histopathologically, with 21 patients in both the experimental and control groups. The basic information of the patients is shown in Table 1.

**Table 1:** Baseline characteristics of the 42 study patients.

Characteristic	No. (%)		
	Experimental group (n = 21)	Control group (n = 21)	Overall (n = 42)
Age, median (range), yr	31.7 (7.5–58.2)	33.1 (8.1–67.0)	42 (7.5–67.0)
Gender			
Male	9 (42.9)	9 (42.9)	18 (42.9)
Female	12 (57.1)	12 (57.1)	24 (57.1)
Cyst location			
Maxilla	9 (42.9)	9 (42.9)	18 (42.9)
Mandible	12 (57.1)	12 (57.1)	18 (42.9)
Affecting structures			
Tooth root	20 (95.2)	19 (90.5)	39 (92.9)
Inferior alveolar neurovascular bundle	12 (57.1)	12 (57.1)	24 (57.1)
Maxillary sinus	6 (26.8)	6 (26.8)	12 (28.6)
Nasal cavity	3 (14.3)	4 (19.0)	7 (16.7)
Permanent tooth germ	1 (4.8)	2 (9.5)	3 (7.1)

### 3.2. Application and effectiveness of the wing-shaped plug

In the experimental group, all the wing-shaped plugs were successfully molded, fabricated, and fitted into the oral cavity, conforming well to the postoperative window and surrounding tissue morphology (Fig. 2F). Our findings indicate that the new wing-shaped plug has superior retention, higher comfort, better aesthetics, no deformation issues, is easy to wear, and does not cause noticeable pain or irritation. The surrounding tissues remained healthy, and there was no significant impact on daily life. In contrast, the traditional drainage tubes had poorer retention, were less comfortable to wear, less aesthetically pleasing, prone to deformation, often caused pain and irritation, and led to tissue overgrowth, inconveniencing daily activities (Table 2).

**Table 2:** Comparison of patient experience between those using the wing-shaped plug and the drainage tube.

Item	Group (%)		χ^2^	*P*
	Control (n = 21)	Experimental (n = 21)		
Tissue-friendly				
Yes	2 (9.5)	21 (100)		
No	19 (90.5)	0 (0)	–	<.001
Good retention achieved				
Yes	14 (66.7)	21 (100)		
No	7 (33.3)	0	–	<.001
Nondeformable				
Yes	6 (28.6)	21 (100)		
No	15 (71.4)	0	–	<.001
Pain-free experience				
Yes	4 (19)	20 (95.2)		
No	17 (81)	1 (4.8)	28.77^a^	<.001
Easily cleanable				
Yes	2 (9.5)	21 (100)		
No	19 (90.5)	0	–	<.001
No impact on daily life				
Yes	5 (23.8)	20 (95.2)	26.19^a^	
No	16 (77.2)	1 (4.8)		<.001

### 3.3. Shortened treatment time with the wing-shaped plug

**Figure 4.**
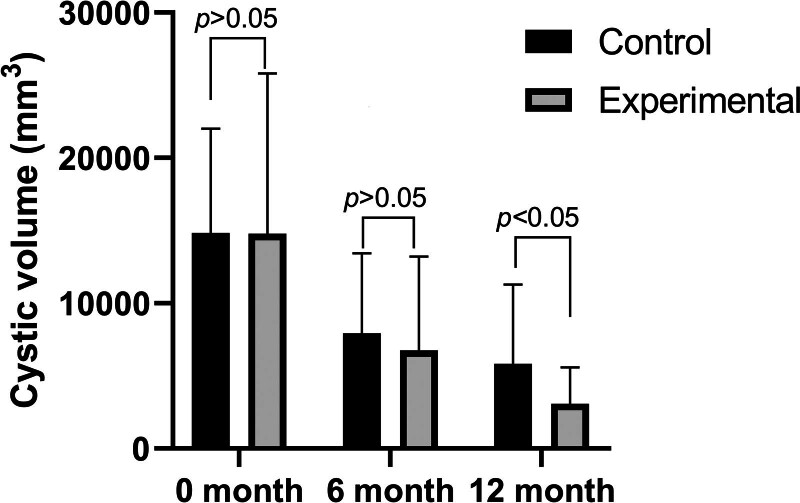
Volumes of cysts in both groups at 0, 6, and 12 months.

**Figure 5.**
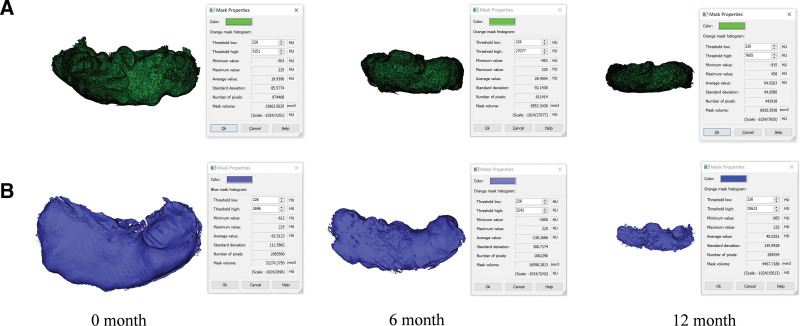
(A) Examples of cyst cavity volume reconstruction in the control group at 0, 6, and 12 months. (B) Examples of cyst cavity volume reconstruction in the experimental group at 0, 6, and 12 months.

**Figure 6.**
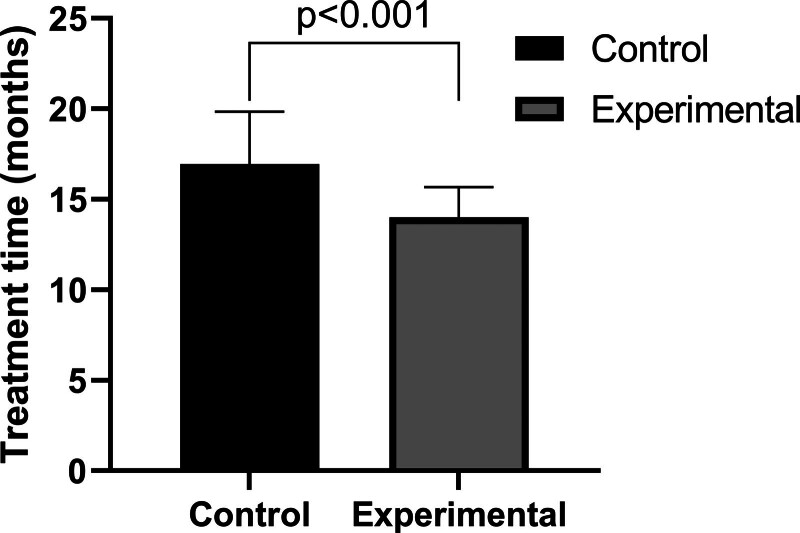
Total duration of treatment required for both groups.

In the experimental group, the cyst cavity showed a reduction in size by the 6th month compared to the control group, though the difference was not statistically significant. However, by the 12th month, the experimental group had a significantly smaller cyst cavity compared to the control group, with a statistically significant difference (Figs. 4 and 5). The total wearing time for the control group (16.95 ± 2.89 months) was significantly longer than for the experimental group (14.00 ± 1.67 months) (*P* < .001). The wing-shaped plug reduced the treatment time by 3 months compared to the traditional drainage tube (Fig. 6).

## 4. Discussion

OKCs primarily involves surgical resection combined with adjuvant therapies.^[11]^ Adjuvant treatment options include Carnoy solution (CS), modified CS, and 5-fluorouracil (5FU). For large cysts, treatment approaches mainly encompass surgical and conservative methods.^[11]^ Literature suggests that a combination of marsupialization and cyst enucleation for medium to large cysts can reduce recurrence rates and transform the cysts into less aggressive lesions. Furthermore, initial marsupialization of OKCs involving critical structures such as blood vessels, nerves, tooth buds, and sinus cavities helps preserve the integrity of surrounding tissues and organs.^[11,12]^ Our study corroborates these findings, demonstrating similar beneficial outcomes.

Leite-Lima^[13]^ and Consolo^[14]^ reported that postmarsupialization, as the cyst volume decreases, the cyst wall becomes more fibrous, and the thin epithelial layer thickens, becoming less fragile and more firmly attached to the cyst wall. They also observed a reduction in epithelial hyperplasia and, in some instances, the disappearance of the typical keratinizing cystic epithelial layer.^[13,14]^ These changes render the cyst tissue more akin to oral mucosa, thereby facilitating surgical resection. Consequently, the histological alterations following marsupialization enhance the effectiveness of cyst treatment.^[13,14]^ Open decompression has been shown to promote bone defect recovery and reduce cyst size, indicating that OKC growth is reversible and reinforcing the efficacy of marsupialization.^[14,15]^ This therapeutic effect may be mediated through the inhibition of IL-1α expression and epithelial cell proliferation, contributing to the reduction in OKC size.^[16]^ Our findings further validate these mechanisms, underscoring the potential of marsupialization as a crucial step in the management of OKCs.

Marsupialization requires the maintenance of an open cavity postoperatively to facilitate structural changes within the cyst. Various decompression devices have been described in the literature. For instance, Haribabu^[17]^ utilized 3.0 silk sutures to secure nasopharyngeal airway tubes to the buccal soft tissues of the odontogenic keratocyst, employing them as flushing devices to keep the cystic cavity patent. While this method is straightforward and convenient, it presents notable drawbacks, including significant irritation, instability, and potential discomfort for the patient. Kolokythas^[8]^ employed stainless steel wires to fix decompression tubes to adjacent teeth. This approach reduces trauma to surrounding soft tissues compared to sutures and provides relatively secure fixation. However, it is not suitable for edentulous patients, who require traditional suture fixation, and there is a risk of tube dislodgement or displacement. These issues were also observed in the control group of our study. To enhance marsupialization techniques and devices, researchers like Kivovics^[18]^ have developed custom, removable dental-supported cyst plugs using digital workflows. Although digital technology improves the accuracy of cyst plugs, it entails higher costs associated with materials, software, and hardware, and requires the presence of remaining teeth for attachment. Consequently, our study explored an alternative approach using molding techniques to create personalized cyst plugs. This method aims to maintain cavity patency while being comfortable, tissue-friendly, and effective, offering a promising new option for improving marsupialization outcomes.

This study demonstrates that the wing-shaped plug significantly reduces the duration of marsupialization compared to traditional drainage tubes. This improvement is likely due to the plug’s ability to maintain effective drainage of the cyst cavity. Traditional drainage tubes often encounter issues such as clogging or dislodgement, which can compromise drainage efficiency and adversely affect the outcome of marsupialization. In contrast, the novel wing-shaped cyst plug, being compact and comfortable, offers enhanced tissue compatibility. This is attributed to the precise molding techniques and muscle function adjustments utilized during its fabrication.^[19]^ The adjustment for muscle function during the molding process ensures a precise fit of the plug’s wings to the vestibular sulcus, similar to the fitting of a removable denture, thereby enhancing patient comfort. Additionally, the plug’s design, with wings being pressed by the labial and buccal muscles and the body positioned within the cyst cavity, contributes to its effective retention during functional oral activities.

The introduction of this new and straightforward method for marsupialization of odontogenic keratocysts demonstrates promising efficacy while offering advantages such as compactness, comfort, and tissue compatibility. Additionally, this approach may be applicable to other types of cysts requiring marsupialization. However, the study has several limitations, including a relatively short follow-up period and a small sample size. To confirm the clinical efficacy and broader applicability of the wing-shaped plug, further research with a larger sample size and extended follow-up period is necessary.

## 5. Conclusion

The novel wing-shaped cyst plug is a simple to fabricate, highly effective, and comfortable tool for marsupialization and decompression. It demonstrates significant clinical potential and shows promising prospects for future application.

## Acknowledgments

We would like to express our gratitude to all staff from the Stomatology Hospital of Guangxi Medical University, for their collecting, verifying and cleaning of the data used in this study.

## Author contributions

**Conceptualization:** Haifeng Liu, Nuo Zhou, Xuan-ping Huang.

**Data curation:** Haifeng Liu, Xuan-ping Huang.

**Formal analysis:** Xuan-ping Huang.

**Funding acquisition:** Xuan-ping Huang.

**Investigation:** Xuan-ping Huang.

**Methodology:** Xuan-ping Huang.

**Project administration:** Nuo Zhou, Xuan-ping Huang.

**Resources:** Nuo Zhou, Xuan-ping Huang.

**Software:** Haifeng Liu, Nuo Zhou.

**Supervision:** Haifeng Liu, Nuo Zhou.

**Validation:** Nuo Zhou.

**Visualization:** Nuo Zhou.

**Writing – original draft:** Haifeng Liu.

**Writing – review & editing:** Haifeng Liu, Nuo Zhou.
